# Clinical characteristics of rhegmatogenous retinal detachment in patients over 90 years in a tertiary center in Germany: 90-TOSG report 2

**DOI:** 10.1007/s00417-025-06821-w

**Published:** 2025-04-11

**Authors:** Focke Ziemssen, Ayşe Güzin Taşlipinar Uzel, Spyridon Dimopoulos, Jonas Neubauer, Karl Ulrich Bartz-Schmidt, Faik Gelisken, 90-Tübingen Ophthalmology Study Group

**Affiliations:** 1 Department of Ophthalmology, University of Leipzig, Leipzig, Germany; 2 Department of Ophthalmology, Haydarpasa Numune Training and Research Hospital, Istanbul, Turkey; 3 Center of Ophthalmology, Eberhard Karl University Tübingen, Tübingen, Germany; 4 Universitätsklinikum Leipzig, Klinik Für Augenheilkunde, Liebigstraße 12, 04103 Leipzig, Germany

**Keywords:** Rhegmatogenous retinal detachment, Demographics, High-age, Proliferative vitreoretinopathy

## Abstract

**Purpose:**

This study aims to investigate the clinical characteristics of rhegmatogenous retinal detachment (RRD) in patients aged over 90 years, a demographic that has been underrepresented in previous research.

**Method:**

Retrospective, single-center, observational case series. The study included patients over 90 years old, excluding those with significant ocular trauma or other specific eye diseases affecting the retina. Data on demographic characteristics, RRD extent, surgical interventions, and postoperative outcomes were collected and analyzed.

**Results:**

The study included 24 patients (24 eyes, 75% female) where the median age was 93 years (range: 91–98). Most patients displayed macular involvement, and about one-third experienced proliferative vitreoretinopathy. Surgical interventions primarily involved pars plana vitrectomy, with a noted delay from symptom onset to surgery averaging ten days. Postoperative improvements in visual acuity were significant, yet the final visual acuity remained low. Baseline median BCVA was 2.30 logMAR, and 1.0 (0.30–2.30) log MAR at the last visit (*p* = 0.017). RRD was in 52% of the eyes over two quadrants, in 83.3% with macula involvement, and in 37.5% with proliferative vitreoretinopathy (PVR) at baseline. Of these patients, 61.9% were pseudophakic. Twenty-one eyes of 21 patients underwent treatment and postoperative follow-up was available in 47.6% (*n* = 10) of the patients. Four of the 10 patients with follow-up had redetachment at the last visit.

**Conclusion:**

The high rate of macular involvement and the complexities associated with PVR highlight the challenges of treating RRD in the elderly. Delayed hospital presentation, impaired adherence to posturing and low postoperative follow-up rates may significantly impact the treatment success. This study underscores the need for tailored management strategies to improve outcomes in this age group.

**Key Messages:**

***What is known***

- An increase in retinal detachments has occurred mainly due to earlier lens surgery in the younger age group but will also occur in the old-aged group as a result of demographic changes.

***What is new***

- Elderly patients show variable symptoms of retinal detachments and management is complicated by frequent ocular and systemic comorbidities.
- The clinical characteristics of detachments at age over 90 years include proliferative vitreoretinopathy and late presentation.
- Like vitreoretinal surgery at the end of life, the decision to operate presents a particular challenge.

## Introduction

As life expectancy and the number of elderly people in the industrialised countries continue to rise and the proportion of seniors in the global population is projected to reach to 22.0 percent by 2050, with one in every 11 individuals expected to be aged 80 or older [1]. The demographic shift emphasizes the need to focus on age-associated health issues, particularly those that impair independence and quality of life [2].

Rhegmatogenous retinal detachment (RRD) is a significant contributor to vision loss [3]. Although more frequent and earlier lens surgery has led to a doubling of the incidence over the recent decade [4, 5], especially in younger age groups, a further sharp increase is seen in old age [6].

In elderly patients, the more variable manifestation of RRD and the increasing number of ocular and systemic concomitant diseases make the management difficult [7, 8].

In the elderly, particularly those over 90, degenerative eye diseases such as age-related macular degeneration (AMD) and glaucoma are prevalent causes of vision impairment [9, 10]. However, high quality population-based studies capturing data on the incidence and progression of RRD with the stratification by age and comorbidities are still missing [3]. Some epidemiological studies show large gaps in or even neglect the older population [5]. The limited access to eye care with increasing need of care is likely to contribute to a large extent to the fact that curves fall more steeply from the age of 90 than the projections suggest [4, 11].

Therefore, this study aims to address this gap by evaluating the clinical characteristics and treatment outcomes of RRD in patients aged over 90 years. By focusing on this unique population, we seek to understand better how age influences the presentation, management, and outcomes of the condition, thereby providing more effective clinical strategies for this growing segment of the population.

## Methods

This retrospective study reviewed medical records of patients aged 90 years and above, diagnosed with RRD from 2003 to 2021 at the Ophthalmology Department of the University of Tübingen. The study protocol of the 90—Tuebingen Ophthalmology Study Group (90-TOSG) was approved by the local ethics committee and adhered to the principles of the Declaration of Helsinki. We selected 90 + years as the cut-off based on established gerontological and epidemiological classifications, recognizing nonagenarians as the "oldest-old." The 2015 WHO Report on Aging and Health emphasizes that aging is influenced by functional ability, and research highlights distinct physiological, cognitive, and functional patterns in nonagenarians [12]. Additionally, late-life mortality deceleration research and Gompertz’s Law suggest a shift in aging dynamics at 90 years, making it a widely accepted threshold for studying extreme aging and its healthcare implications [13].

Patients were excluded if they had a history of penetrating or ruptured globe trauma, uveitis, ocular ischemic syndrome, or RRD with tractive components secondary to proliferative diseases. This approach ensured a homogeneity in the study population that was necessary for valid comparisons.

Demographic data, concomitant ophthalmic and systemic diseases, medications, and RRD characteristics were systematically collected. We also recorded the surgical techniques used, type of the anesthesia, complications during the surgery, duration of the hospitalization, and number of the postoperative visits. For each patient, the extent of RRD was evaluated based on quadrant involvement, with specific attention to macular involvement.

All patients underwent a complete ophthalmological examination including slit-lamp fundoscopy using a Goldmann 3-mirror lens. Best-corrected visual acuity (BCVA) was measured using Snellen charts and converted to the Logarithm of the Minimum Angle of Resolution (LogMAR) scale for analysis. The extent of RRD was evaluated as quadrant involvement. Involvement of the macula was also noted. Only patients who had at least one month of follow-up data after surgery were included in the outcome analysis.

Data analysis was performed using SPSS Statistics 21 (IBM Corp, Armonk, NY, USA). Results were presented as medians and ranges, with categorical data expressed as percentages. The Wilcoxon signed-rank test was used to evaluate dependent variables. Statistical significance was established at a p-value of less than 0.05.

## Results

A total of 24 eyes of 24 patients with RRD were analyzed. The median age was 93 years (range: 91–98), with a predominance of female patients (*n* = 18, 75%). RRD was equally observed in the right and left eyes (50% each). The median time from symptom onset to hospital admission was nine days (range: 2–120).

Baseline BCVA was median 2.30 logMAR (range:0,40–3.00; Snellen equivalent range: 20/50 to hand movement). Lens status showed that 33.3% were phakic, while 61.9% were found to be pseudophakic and 4.7% aphakic. Common comorbidities included glaucoma (12.5%), age-related macular degeneration (AMD) (8.3%), and ectropion (4.2%). Notably, 37.5% of patients were on anticoagulant or antiplatelet therapy (Table 1).

**Table 1:** Systemic diseases of patients with rhegmatogenous retinal detachment

	*n*, (%)
Systemic hypertension	17 (70.8)
Diabetes mellitus	5 (20.8)
Heart insufficiency	4 (16.7)
(Previous) Myocardial infarction	2 (8.3)
Presence of pacemaker	3 (12.5)
Coronary artery disease	1 (4.2)
Hypercholesterolemia	3 (12.5)
Dysrhythmia	3 (12.5)
(Previous) Stroke	3 (12.5)
Pulmonary embolism	2 (8.3)
Deep vein thrombosis	1 (4.2)
Varicosis veins	2 (8.3)
Chronic kidney failure	2 (8.3)
Dementia	3 (12.5)
Psychosis	1 (4.2)
Hypothyroidism	3 (12.5)
Hyperthyroidism	1 (4.2)
Osteoporosis	2 (8.3)
Arthrosis	2 (8.3)
Benign prostatic hyperplasia	2 (8.3)

One patient had previously undergone detachment surgery in the same eye, and two patients had a history of non-penetrating ocular trauma (8.3%).

Median BCVA of the fellow eye was 0.40 logMAR (range: 0–2.70). The prevalence of glaucoma and AMD was 12.5% and 33.3% for the fellow eyes, respectively. Almost half of the unaffected eyes were pseudophakic, similar to the affected eye.

## Extent of detachment and surgical interventions

RRD affected in average of two quadrants per eye, with 43.4% (*n* = 10) of eyes showing involvement in all four quadrants. One quadrant was involved in 34.7% (*n* = 8) of the eyes, two quadrants in 13% (*n* = 3), three quadrants in 8.6% (*n* = 2). The extent of RRD was not noted for one eye.

Macular involvement was found in 83.3% (*n* = 20) of the eyes, indicating a high risk of vision loss at baseline. In 8.3% (*n* = 2) of the eyes RRD was accompanied by vitreous hemorrhage, and 37.5% (*n* = 9) of the eyes had proliferative vitreoretinopathy (PVR).

The median time interval from hospital admission to surgery was one day (range: 0–23).

Pars plana vitrectomy (PPV) was the most common procedure (71.4%), followed by scleral buckling (14.2%) and a combination of both (14.2%). Two patients were considered not suitable for the retinal detachment surgery because of the poor visual prognosis in presence of comorbidities of late-stage AMD and advanced PVR. One patient refused surgery.

PPV was combined with cataract surgery in 23.8% (*n* = 5) of the eyes. C_2_F_6_ was used as endotamponade in 44.4% (*n* = 8) of the eyes, C_3_F_8_ in 11.1% (*n* = 2) and silicone oil in the remaining 44.4% (*n* = 8).

The majority of surgeries were performed under parabulbar anesthesia (81%). The mean duration of the operation was 45 min (range: 15–115). The mean duration of hospitalization was 4 days (range: 3–9).

Complications included mild vitreous hemorrhage in 11.1% of the eyes, gas leakage 4.7% and subchoroidal hemorrhage 4.7%.

No retinal break was found in 25% (*n* = 6) of the eyes. In 37.5% (*n* = 9) of the eyes the retinal break was in the upper half of the retina and in 29.1% (*n* = 7) in the lower half. In one eye (4.1%) the retinal breaks were in the upper and lower half of retina. In one eye (4.1%) full thickness macula foramen was noted.

Follow-up data were available only for 47.6% (*n* = 10) of patients, and showed an improvement in median BCVA of 1.2logMAR (range −0.25 to 1.9) (*p* = 0.017) (Table 2). Despite this improvement, persistent RD necessitated reoperations in two eyes one-month postoperatively. The median number of postoperative visits was one (range: 1–8). Silicone oil was removed in one of the seven eyes, 6 months postoperatively.

**Table 2:** Clinical characteristics of patients with follow-up

	Age	Gender	Eye	BCVA^b^ (logMAR)	Concomitant eye disease	Extent of RRD*	Macula off/on	PVR	Procedure	Complication	Follow up time (months)	Final outcome	BCVA^f^ (logMAR)
1	95	m	OS	2.3	glaucoma	1	off	no	SB, PPV, C_2_F_6_, laserpexy	no	1.6	RA	0.7
2	96	f	OD	0.5	amd	1	on	no	PPV,silicone oil, laserpexy	decalin leakage	6	RA	0.7
3	94	m	OS	2.3	no	2	off	yes	PPV, silicone oil, laserpexy	after 3 months silicon emulsification	3	RA	0.3
4	91	f	OS	2.7	high myopia	4	off	yes	PPV, C_2_F_6_	no	1	RD	2.3
5	93	m	OS	1.9	no	2	off	no	SB, 2nd procedure: PPV, silicone oil, laserpexy	no	9	RA	0.5
6	95	m	OD	2.3	no	n.d	on	no	PPV, C_2_F_6_, cryopexy	no	3	RA	0.4
7	93	f	OD	2.3	glaucoma	4	off	no	PPV, silicone oil, laserpexy	no	1	RD	2.3
8	95	m	OS	2.3	hemianopia	4	off	yes	SB, PPV, silicone oil	no	3	RA	2.3
9	93	f	OS	2.7	no	2	off	no	PPV, C_2_F_6_, laserpexy 2nd procedure: PPV, silicone oil, laserpexy	subchoroidal hemorrhage	37.6	RD	1.9
10	93	f	OS	2.7	no	4	off	no	PPV, silicone oil, laserpexy	no	2	RD	1.3

*m* male, *f* female, *OD* right eye, *OS* left eye, *BCVA*^*b*^ baseline best corrected visual acuity, *n.d.* not detected, *AMD* Age related macular degeneration, *PVR* proliferative vitreoretinopathy, *PPV* pars plana vitrectomy, *SB* scleral buckling, *RA* reattachment, *PD* persistent detachment, *RD* redetachment, *BCVA *^*f*^ final best corrected visual acuity

^*^The extent of RRD in involved quadrants

## Discussion

The study highlights that macular involvement and PVR are common in patients over 90 years with RRD. Despite advances in surgical techniques leading to significant improvements in visual acuity, the study points to persistent barriers related to delayed presentation and low postoperative follow-up rates that significantly impact the overall success of treatment outcomes in this elderly patient group.

Despite significant improvements, the final median BCVA remained low at 1.0 logMAR (Snellen equivalent 20/200), reflecting the challenges in managing advanced RRD in this age group. Our findings corroborate previous studies noting a female predominance in older RRD patients, likely due to longer life expectancy in women [5, 9, 10]. The postoperative follow-up rate was markedly low, which could partially be explained by local follow-ups outside of tertiary care settings. However, the low anatomical success rate (60% among those followed) underscores the need for improved management and follow-up strategies. Elderly patients may face challenges in adhering to posturing requirements due to musculoskeletal limitations and cognitive changes, potentially impacting surgical outcomes [14–16]. However, the limited number of very old patients included in studies on objective posturing assessment after vitreoretinal surgery restricts the ability to fully evaluate the impact of age [17, 18]. This study expands on existing literature by detailing the complications specific to an older demographic, where cognitive impairments and physical limitations further complicate timely access to care. In patients over 90, surgery should not be routine but considered on a case-by-case basis, with shared decision-making that may include withholding intervention when risks outweigh benefits [19].

Of note was the short follow-up period and the low anatomical success rate among the followed patients (47.6%). Although the functional improvement was significant, the median final visual acuity was relatively low with 20/200. The effect of the other ocular comorbidities should also be considered when interpreting the outcome in successfully treated elderly patients [7, 20].

The extended time from the symptom onset to hospital admission may have significantly influenced the surgical outcomes. Delays, often longer in the very elderly due to logistic and cognitive challenges, increase the risk to macula-off RRD progression [21]. Limited awareness and barriers such as instutionalozation or physical disabilities further hinder timely access to specialist care [22–24]. Duration of RRD remains to be an important, at least partially modifiable risk factor [9]. While the median time between the onset of symptoms and presentation was nine days in the current study in other studies, previous reports in patients between 70–91 years indicate a shorter delay of 4–5 days [7, 8]. To better understand the extreme delays, we reviewed the case of the 120-day outlier, a patient with a history of ocular disease but no evident external barriers such as hospitalization, comorbidities, or transportation issues. This raises concerns about whether prior healthcare interactions promoted awareness and if cognitive decline or passive health-seeking contributed to delayed diagnosis [21].

Beyond age, the large extent and prolonged duration of RRD in elderly patients are additional risk for PVR development [25, 26]. In our study, PVR was present in 37.5% of the eyes, with 87.5% involving RRD in two or more quadrants, while the median time from the symptoms to the surgery was 10 days. The unexpectedly high rate of buckle-only and combined procedures in our cohort likely reflected the careful selection of surgical approaches. While one might expect combined surgery to be more common, factors such as the preservation of lens status, the surgeon’s preference to minimize intraocular intervention in vulnerable elderly patients, and the importance of achieving single-procedure success likely contributed to this observation. The absence of a clear-cut guideline for this age group and the still significant proportion of phakic eyes might further support the rationale for this approach. The absence of short-acting gas use in this cohort may reflect surgeon preference, though its potential benefits, such as reducing binocular vision disturbance and expediting visual recovery, warrant further consideration.

Our study detected macular involvement in 83.3% of cases, higher than the 69% observed in younger cohorts aged 70 to 87 years [10]. Older patients are more likely to present with macula involvement after RRD than younger patients [7]. Pseudophakia is known as an independent risk factor for RRD with macula involvement [27]. With the even lower rate of pseudophakia of this study, the rate of macula involvement was striking and needs further confirmation. The presence of superior retinal tears is a risk factor for converting macula-on to macula-off RRD while waiting for surgery [28]. Although the rate of inferior tears increases with age, in the elderly population, retinal tears are most commonly seen in the superior retina [29, 30]. Even if no certain retinal tears were detected in 25% of the eyes, a superior retinal location cannot be excluded. The presence of superior retinal tear and late presentation may be responsible for high macular involvement in the present study. The observation might point to tailored approaches of RRD management.

The incidence of PVR, an important risk factor for the success of the surgical outcomes, was also high. The relationship between extensive detachment, delayed treatment, and increased PVR prevalence aligns with the literature and emphasizes the complexity of treating RRD in the elderly.

A significant limitation of our study is the low number of postoperative visits, which may not solely be attributed to the patients’ advanced age, as this issue is less reported in similar-aged cataract surgery patients. This suggests that factors specific to RRD, such as slow visual recovery and limited mobility, might deter follow-up. Facing the low numbers, no conclusive statement can be made of the total rate of the anatomical success. Future studies should explore strategies to enhance follow-up compliance and assess long-term outcomes more effectively. Additionally, the retrospective nature of our study and the small sample size limit the generalizability of our findings and highlight the need for prospective studies in this field.

Managing RRD in patients over 90 is challenging due to high rates of macular involvement, PVR, and postoperative complications. Enhancing early detection and rapid treatment, improving follow-up rates, and tailoring surgical approaches to the specific needs of the elderly can potentially improve outcomes in this vulnerable population.
